# P-1288. Changes in Social Contact Patterns Concerning the Respiratory Illness: A Chronological Study in Japan

**DOI:** 10.1093/ofid/ofae631.1469

**Published:** 2025-01-29

**Authors:** Hwichang Jeong, Sehyun Park, June Young Chun, Yongdai Kim, Shinya Tsuzuki

**Affiliations:** Seoul National Univerisity, SEOUL, Seoul-t'ukpyolsi, Republic of Korea; Seoul National University, seoul, Seoul-t'ukpyolsi, Republic of Korea; National Cancer Center, Goyang, Kyonggi-do, Republic of Korea; Seoul National University, seoul, Seoul-t'ukpyolsi, Republic of Korea; National Center for Global Health and Medicine

## Abstract

**Background:**

Transmission dynamics of respiratory pathogens are largely dependent on social mixing patterns, Besides, individuals who are ill might change their contact behavior according to their symptoms. However, there is a paucity of data on social mixing patterns concerning the respiratory illness.

**Figure:**
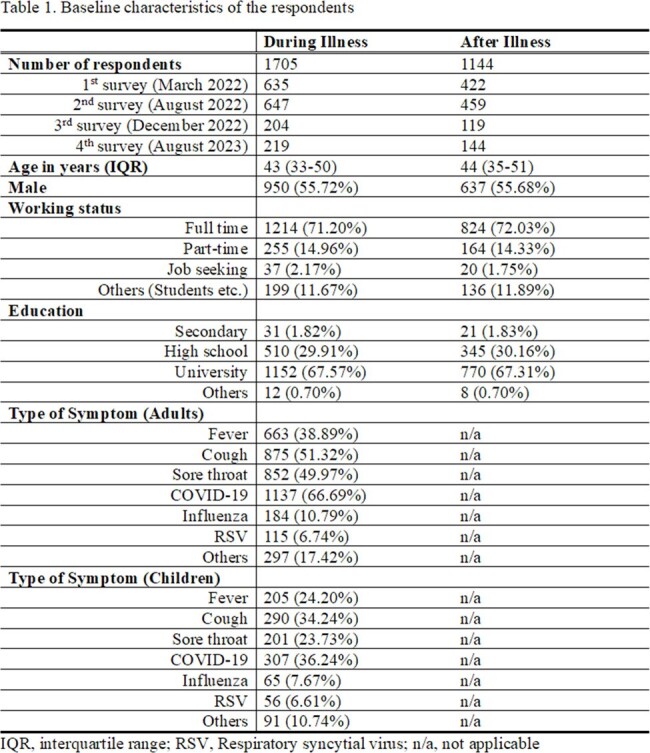

**Methods:**

Here, we conducted an internet-based periodic survey in Japan since 2022 to characterize the differences in mixing patterns during and after the illness. First, participants who had the respiratory illness were recruited and followed up 2 weeks later.

Contact matrices consisting of the age-specific average number of contacted persons recorded per day were obtained from the survey data, using the bootstrap method. Reciprocity was achieved by using the weighted averages according to the age-specific population sizes [1].

**Figure:**
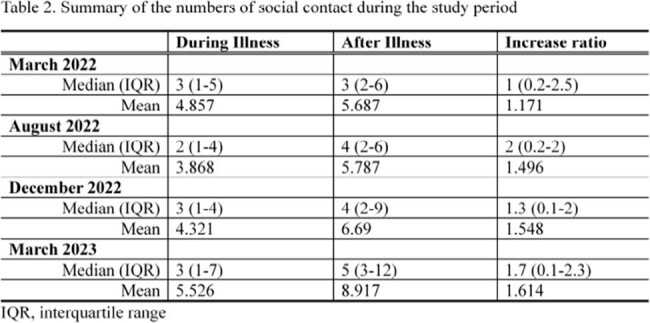

**Results:**

Contact patterns were generally age-assortative, and the average contact numbers had gradually increased from March 2022 to August 2023. Most recently, the median number of contacts per day during respiratory illness was 3 (interquantile range [IQR] = 1-7) and then rose to 5 (IQR = 3-12) in August 2023. The increase ratio was 1.7 (IQR = 0.1-2.3). The baseline frequency of social contacts during illness was identical to the numbers studied in 2021 in Japan (median number of contacts = 3 [IQR = 1-6]) [2]. The number of contacts increased after illness but considering the contact frequency in the 2011 study (median number of contacts = 12), it has not yet reached the pre-COVD-19 status [3].

**Figure 1.**
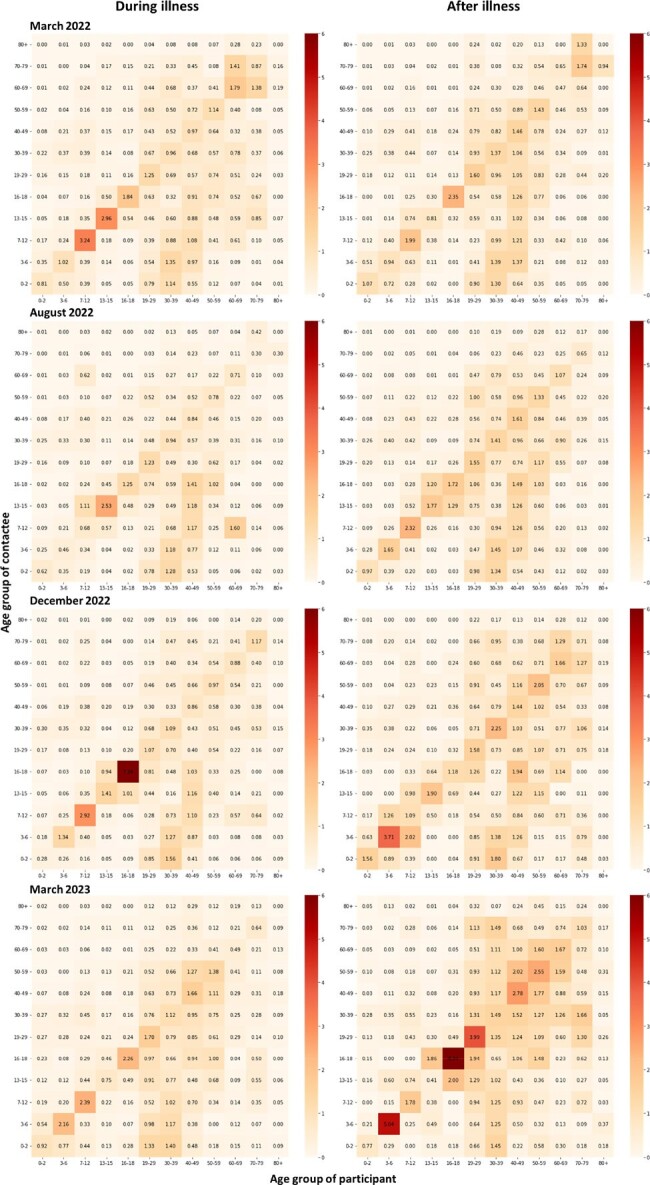
Contact matrices during and after illness within the study period.

**Conclusion:**

In this study, we found that individuals tend to decrease their contacts by up to 50% when they feel unwell. This gap was not found until March 2022 in Japan when social distancing measures against COVID-19 remain. Afterward, the frequency of social contact tends to rise but has not yet reached the pre-COVID-19 era as of August 2023.

**Disclosures:**

**All Authors**: No reported disclosures

